# Epigenetic Regulation via Altered Histone Acetylation Results in Suppression of Mast Cell Function and Mast Cell-Mediated Food Allergic Responses

**DOI:** 10.3389/fimmu.2018.02414

**Published:** 2018-10-23

**Authors:** Dylan Krajewski, Edwin Kaczenski, Jeffrey Rovatti, Stephanie Polukort, Chelsea Thompson, Catherine Dollard, Jennifer Ser-Dolansky, Sallie S. Schneider, Shannon R. M. Kinney, Clinton B. Mathias

**Affiliations:** ^1^Department of Pharmaceutical and Administrative Sciences, College of Pharmacy and Health Sciences, Western New England University, Springfield, MA, United States; ^2^Northampton High School, Northampton, MA, United States; ^3^Baystate Medical Center, Pioneer Valley Life Sciences Institute, Springfield, MA, United States

**Keywords:** mast cells, food allergy, trichostatin A, histone deacetylase (HDAC) inhibitors, epigenetics

## Abstract

Mast cells are highly versatile cells that perform a variety of functions depending on the immune trigger, context of activation, and cytokine stimulus. Antigen-mediated mast cell responses are regulated by transcriptional processes that result in the induction of numerous genes contributing to mast cell function. Recently, we also showed that exposure to dietary agents with known epigenetic actions such as curcumin can suppress mast cell-mediated food allergy, suggesting that mast cell responses *in vivo* may be epigenetically regulated. To further assess the effects of epigenetic modifications on mast cell function, we examined the behavior of bone marrow-derived mast cells (BMMCs) in response to trichostatin A (TSA) treatment, a well-studied histone deacetylase inhibitor. IgE-mediated BMMC activation resulted in enhanced expression and secretion of IL-4, IL-6, TNF-α, and IL-13. In contrast, pretreatment with TSA resulted in altered cytokine secretion. This was accompanied by decreased expression of FcεRI and mast cell degranulation. Interestingly, exposure to non-IgE stimuli such as IL-33, was also affected by TSA treatment. Furthermore, continuous TSA exposure contributed to mast cell apoptosis and a decrease in survival. Further examination revealed an increase in I-κBα and a decrease in phospho-relA levels in TSA-treated BMMCs, suggesting that TSA alters transcriptional processes, resulting in enhancement of I-κBα transcription and decreased NF-κB activation. Lastly, treatment of wild-type mice with TSA in a model of ovalbumin-induced food allergy resulted in a significant attenuation in the development of food allergy symptoms including decreases in allergic diarrhea and mast cell activation. These data therefore suggest that the epigenetic regulation of mast cell activation during immune responses may occur *via* altered histone acetylation, and that exposure to dietary substances may induce epigenetic modifications that modulate mast cell function.

## Introduction

IgE-mediated mast cell activation plays a critical role in the development of allergic responses to food antigens (1, 2). Mast cells and their mediators drive acute episodes of food allergy resulting in the development of severe intestinal anaphylaxis, which is often manifested as diarrhea, shock, and painful abdominal cramps. Furthermore, the effects of mast cells are mediated through a complex interplay of cellular interactions involving allergen-specific Th2 cells and other cell types such as eosinophils, epithelial cells, and ILC2s, which together contribute to the development of acute intestinal inflammation underlying the clinical symptoms (2–4).

The incidence of food allergy in the West has been exponentially rising and approximately 3–6% of individuals manifest food allergy symptoms (5, 6). However, not everyone undergoes allergic sensitization to the same allergen and allergic children are often able to outgrow some of the allergies they previously exhibited. Although the cells and molecules mediating allergic reactions have been well-studied, the mechanisms underlying the regulation of allergic sensitization and immune activation are still poorly understood. Accumulating evidence from a number of studies suggests that the development of the allergic response is tightly regulated via a complex network of interactions between immune cells, genes, and the environment that result in the inhibition of tolerance mechanisms and the promotion of allergic sensitization to environmental allergens (2, 7). Both genetic polymorphisms and exposure to various environmental stimuli have been shown to increase the susceptibility of developing allergic disease. With respect to the latter, colonizing microbiota, history of prior infections, dietary components, and exposure to environmental factors such as pollution or antibiotic treatment have all been demonstrated to shape the outcome of the allergic response (2, 6–13)). None of these variables by themselves however can account for differences in allergic sensitization in diverse patient subsets, suggesting that the induction of immune activation may be finely regulated *via* subtle epigenetic interactions involving environmental components and immune genes.

Several types of chromatin epigenetic modifications have been shown to influence gene expression (14). These include methylation of DNA at CpG islands or various post-translational modifications of histone tails, such as acetylation and methylation, resulting in enhanced or decreased access of transcriptional factors to gene promoters or enhancers. The role of epigenetic modifications in driving T cell differentiation and development has been well-established (15–19). Several studies also suggest a role for epigenetic modulation of allergic sensitization and inflammation (18, 20–27). However, the effects of epigenetic modification in modulating the behavior of T cells and particularly mast cells during allergic responses to food antigens has not been extensively examined. We previously demonstrated that frequent ingestion of curcumin, which is an active ingredient of the curry spice turmeric, modulates intestinal mast cell function and suppresses the development of mast cell-mediated food allergic responses, suggesting that exposure to dietary components can regulate the development of food allergy (28). This is especially interesting since a number of people worldwide consume curcumin on a daily basis and it has been shown to have immunomodulatory properties, which influence the activation of immune cells. Recent studies further suggest that the effects of curcumin may be mediated via regulation of epigenetic modifications that enhance or inhibit inflammatory responses (29–31). We therefore hypothesized that mast cell function during food allergy may be epigenetically regulated resulting in the development or suppression of allergic reactions.

In order to examine the effects of epigenetic regulation of mast cells, we used the well-established histone deacetylase (HDAC) inhibitor Trichostatin A (TSA). TSA, a fungal antibiotic, belongs to a class of extensively studied histone deacetylase inhibitors that have been used to examine epigenetic interactions involving histone acetylation (32–36). The addition of acetyl groups at lysine residues in histone molecules by histone acetyl transferases (HATs) is generally thought to increase DNA accessibility and promote gene expression. In contrast, HDACs remove the acetyl groups, thereby increasing chromatin compaction and inhibiting gene transcription. TSA is a pan-HDAC inhibitor (HDACi), inhibiting the enzyme activity of several class I and class II HDACs, including HDAC 1, 2, 3, 4, 6 and 10 isoforms (37). As such, treatment with pan-HDACi's such as TSA can induce hyperacetylation of histone molecules, with the potential to enhance gene expression (38). Furthermore, they can also directly modulate the activity of non-histone proteins including transcription factors and cell cycle proteins (39, 40). However, depending on the type of immune cell and antigen treatment, both pro- and anti-inflammatory effects have been observed, suggesting that HDAC inhibition can affect the activation of multiple genes both upstream and downstream of the target molecule being examined (32, 34, 41–44). This includes immunomodulatory effects involving NF-κB (45–47), as well as the production of pro-inflammatory cytokines by antigen-exposed immune cells such as macrophages and ILC2s (48–51). Similarly, TSA-mediated suppression of both adaptive and innate allergic airway inflammation has also been observed in mouse models (51–58).

Here, we show for the first time, that treatment with TSA attenuates IgE-mediated mast cell activation during food allergy responses. Balb/c mice sensitized and orally challenged with chicken egg ovalbumin (OVA) develop robust allergic responses including allergic diarrhea, intestinal mast cell activation and the induction of Th2 responses. In contrast, the development of intestinal anaphylaxis and mast cell activation was significantly attenuated in TSA-treated mice. Similarly, Th2 cytokine production and gene expression was also affected in TSA-treated animals. *In vitro* examination of TSA treatment on IgE-activated mast cells demonstrated a significant inhibition of the production of proinflammatory cytokines such as IL-6, IL-13, TNF-α, and IL-4. This was accompanied by decreased mast cell degranulation and FcεRI expression. TSA treatment also modulated mast cell responses to IL-33 stimulation, demonstrating that the effects of TSA are not limited to the IgE signaling pathway. Further examination revealed decreased NF-κB activation in TSA-treated mast cells, suggesting that exposure to TSA alters transcriptional processes regulating NF-κB activation. Taken together, our data elucidate a novel role for TSA in modulating mast cell function during food allergy, suggesting that the activation and function of mast cells is epigenetically regulated.

## Materials and methods

### Animals

Balb/c mice were purchased from Taconic Farms (Germantown, NY, United States) and bred in house. All research was approved by the Institutional Animal Care and Use Committee (IACUC) of Western New England University and was conducted according to IACUC guidelines. Animals used for research were sacrificed using compressed CO_2_ gas.

### Food allergy sensitization and challenge protocol

To induce food allergy, Balb/c mice were intraperitoneally immunized with 50 μg chicken egg ovalbumin (OVA) and 1 mg alum on days 0 and 14 of the experimental protocol as previously described (28, 59). Both OVA and alum were obtained from Sigma Aldrich. Two weeks after the second OVA sensitization, mice were challenged intragastrically with 50 mg OVA every other day for a total of 6 challenges. One hour following the sixth challenge, mice were sacrificed and the development of food allergy was assessed.

### TSA administration

Starting 1 day before the challenge phase of the experiment 75 μg or 2.5 mg/kg by weight of TSA (Sigma Aldrich) in phosphate buffered saline (PBS) was administered intraperitoneally to Balb/c mice which had received OVA sensitization. Administration was continued daily while OVA challenges were performed on alternating days. TSA was also administered to Balb/c mice which had received OVA sensitization but did not receive OVA challenges.

### Treatment with curcumin

The effects of curcumin (Sigma Aldrich) on BMMCs were examined as previously described (28). Briefly, cells were treated with vehicle or 30 μM curcumin in DMSO for varying periods of time (1 and 24 h, respectively) and cells and supernatants were collected for mRNA analysis and assessment of cytokine secretion.

### Measurement of intestinal anaphylaxis

Intestinal anaphylaxis of challenged mice was assessed by scoring the percentage of mice exhibiting allergic diarrhea as previously described (28, 59). Briefly, mice were observed for the presence of diarrhea for 1 h following the sixth challenge and scored as positive or negative for the presence of diarrhea.

### Histological analysis and enumeration of mast cells

Intestinal mast cells were enumerated, as previously described (28, 59). Tissue sections were stained with chloroacetate esterase (CAE) and mast cells were counted in complete cross sections of jejunum.

### Quantitative PCR analysis and ELISAs

Quantitative real-time PCR was performed using Taqman probes (Life Technologies) as previously described (28, 59). Expression of IL-4, IL-5, IL-6, IL-10, IL-13, IL-17, IL-33, IFN-γ, and NF-κB was calculated relative to GAPDH transcripts. ELISAs for murine mast cell protease (mMCP-1) (Life Technologies) and OVA-specific IgE were performed on serum samples taken immediately after sacrifice as previously described (28, 59). ELISAs for IL-4, IL-5, IL-6, TNF-α, and IFN-γ (Biolegend) as well as IL-13 (Affymetryx) were performed on cell supernatants according to manufacturer's instructions as previously described (28, 59).

### Mesenteric lymph node stimulation

Mesenteric lymph node (MLN) cells were harvested from animals after sacrifice and cultured with complete RPMI medium, 200 μg/mL OVA or anti-CD3 (0.2 μg/ml) and anti-CD-28 (0.2 μg/ml) for 4 days. Anti-CD3 and anti-CD-28 were obtained from Biolegend. Cytokines were enumerated in supernatants, as previously described (28, 59).

### Bone marrow-derived mast cell (BMMC) culture

BMMCs were generated from Balb/c mice as previously described (28, 59). Briefly, bone marrow was obtained from the femurs of naïve mice and cultured with 10 ng/mL rIL-3 and rSCF (Shenandoah Biotechnology) in complete RPMI medium (Life Technologies) for 4 weeks prior to experimentation.

### *In vitro* studies with TSA

One million BMMCs per mL were cultured in triplicate with IL-3 and SCF or 2 μg/mL DNP-IgE (Sigma Aldrich). To determine the effects of TSA on proliferation and cytokine production TSA in dimethyl sulfoxide (DMSO) was added in concentrations of 10, 30,100, 300, or 500 nM for varying time points. Control wells were treated with vehicle alone. Cells were then stimulated with 200 ng/mL DNP-BSA (Sigma Aldrich) or 20 ng/mL rIL-33 (Biolegend).

### β-hexosaminidase assay

BMMCs were cultured with rIL-3 and rSCF in the presence or absence of 500 nM TSA for 1 h or overnight. Cells were activated and supernatants and cell lysates were collected 1 h later. β-hexosaminidase (β-hex) activity was assessed, as previously described (28, 59). Briefly, cells were washed and supernatants and pellets were collected. Pellets were lysed with 0.5% Triton X-100. Both supernatants and pellets were then treated with 4-nitrophenyl-N-acetyl-β-D-glucosaminide (p-NAG) substrate (Sigma) for 1 h. Plates were read at 405 nm using a spectrophotometer to determine β-hexosaminidase activity. Data are depicted as percent specific release according to the following formula: (Stimulated supernatants/(supernatant ± pellet)^*^100−unstimulated supernatants/(supernatant ± pellet)^*^100).

### Flow cytometry

BMMCs were incubated with monoclonal antibodies for c-Kit, FcεRI, annexin V and IgE conjugated to either APC, FITC or PE (Biolegend). All antibodies were diluted 1:200 prior to incubation for 20–30 min. Flow cytometry and analysis was performed using an Accuri C6 cytometer and Flowjo software.

### Intracellular cytokine staining

Peritoneal cells were isolated by peritoneal lavage from naïve Balb/c mice. One million cells/mL were treated with or without DNP-IgE and TSA as described above and activated with DNP-BSA. 3 μg/mL of Brefeldin A (Thermofisher Scientific) was added to all samples according to manufacturer's instructions. Six hours later, cells were surface stained for mast cells using c-Kit APC and FcεRI/IgE-FITC. They were then treated for 10 min with fixation reagent (Thermofisher Scientific) followed by washing with 1X Permeabilization buffer (Thermosfisher Scientific). Intracellular cytokines were assessed by staining with IL-13, IL-6, and TNF-α conjugated to PE (Biolegend).

### Western blot

BMMCs were cultured with rIL-3 and rSCF in the presence or absence of 500 nM TSA and DNP-IgE for 1 h or overnight prior to activation with DNP-BSA. After addition of antigen, cells were incubated for 6–8 h. Whole cell extracts were then obtained using RIPA buffer containing 1% Triton X-100 and quantified with Coomassie Plus (Bradford) Protein Assay (ThermoFisher Scientific). Equal amounts of protein were loaded onto 10% SDS-PAGE gels and transferred to PVDF membrane. Membranes were blocked for 1 h in 5% milk or BSA and incubated overnight with primary antibodies [phospho-relA (1:500) and β-actin (1:5,000)]. Antibodies were obtained from Santa Cruz Biotechnology and Abcam, respectively. Membranes were then washed with PBS tween 20 and incubated with the appropriate secondary antibodies. Membranes were washed once again before the addition of chemiluminescent reagent (Invitrogen). Membranes were imaged using a Biorad Chemidoc.

### Statistical analysis

Data are expressed as mean ± SEM, unless stated otherwise. Statistical significance comparing different sets of mice (between 2 groups) was determined by the Student's *t*-test. *p* < 0.05 were considered significant. Analysis was performed using GraphPad Prism software and/or Microsoft Excel.

## Results

### TSA treatment modulates cytokine production in bone-marrow derived mast cells

Mast cells are highly versatile cells that perform a variety of functions depending on the immune trigger, context of activation and cytokine stimulus. Antigen-mediated mast cell responses are regulated by transcriptional processes that result in the induction of numerous genes contributing to mast cell function. To examine the effects of TSA treatment on resting mast cell function, BMMCs were treated with TSA for 24 h in the presence of rIL-3 and rSCF and the expression and secretion of mast cell cytokines was examined.

**Figure 1:**
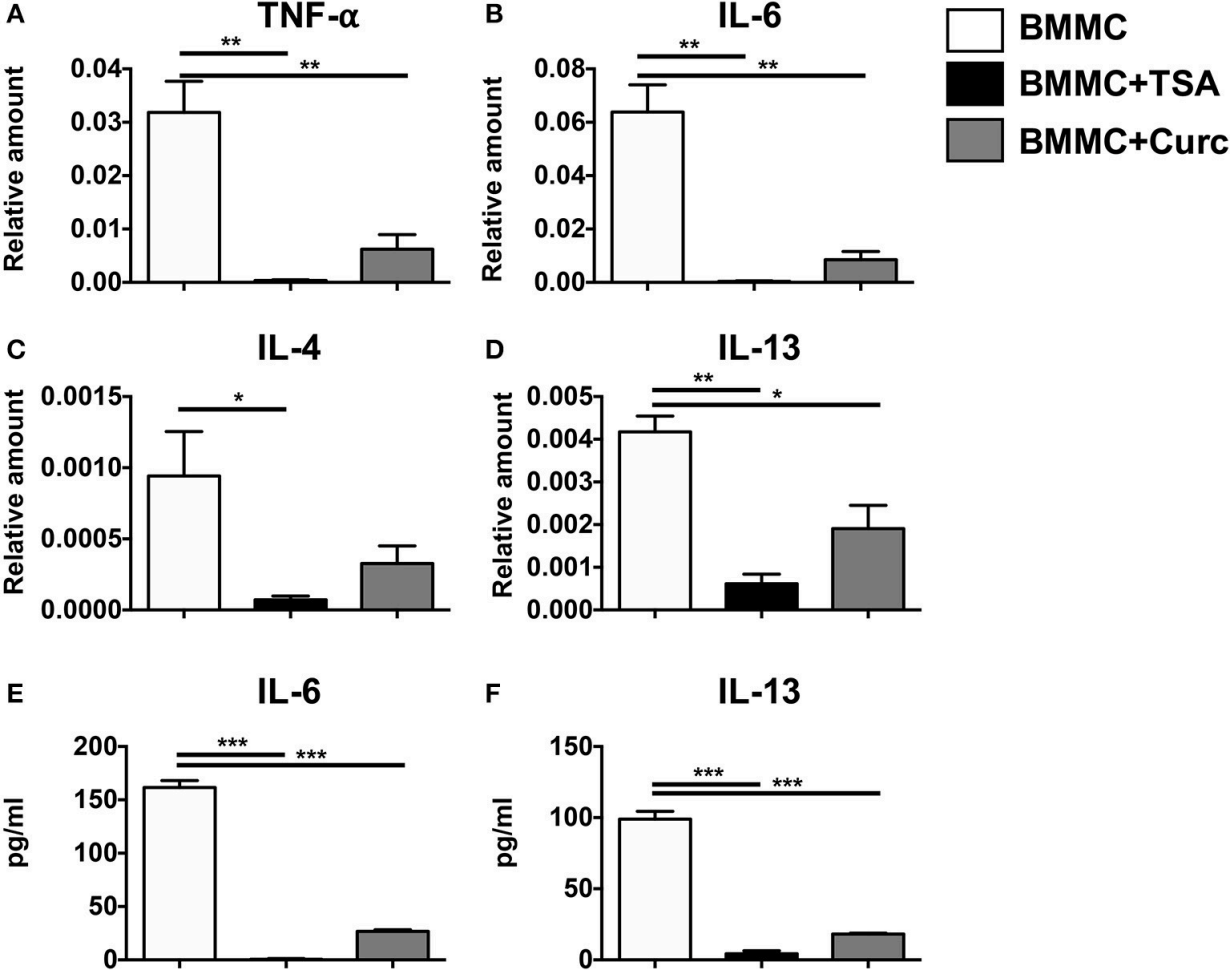
TSA suppresses cytokine gene expression and secretion in resting mast cells. BMMCs were treated with 500 nM TSA, 30 μM curcumin, or vehicle in triplicates. 1 and 24 h later, respectively, cells were collected for mRNA analysis and supernatants were evaluated for cytokine secretion. **(A–D)** mRNA transcripts relative to GAPDH are shown **(E,F)** Levels of IL-6 and IL-13 are shown. Data are representative of 2–3 experiments. *p < 0.01; **p < 0.001; ***p < 0.0001 by Students *t*-test.

Treatment of resting mast cells with rIL-3 and rSCF for 24 h induced the transcriptional upregulation of TNF-α, IL-6, IL-4, and IL-13 (Figures 1A–D). This was also accompanied by the secretion of IL-6 and IL-13 into culture supernatants (Figures 1E,F). In contrast, addition of TSA along with the mast cell growth factors suppressed the expression and secretion of these cytokines, suggesting that TSA suppresses mast cell cytokine production in resting mast cells (Figures 1A–F). A similar inhibition of cytokine production was observed in curcumin-treated mast cells as we have previously demonstrated (Figures 1A–F) (28).

### Pretreatment with TSA inhibits cytokine production in IgE-activated mast cells

**Figure 2:**
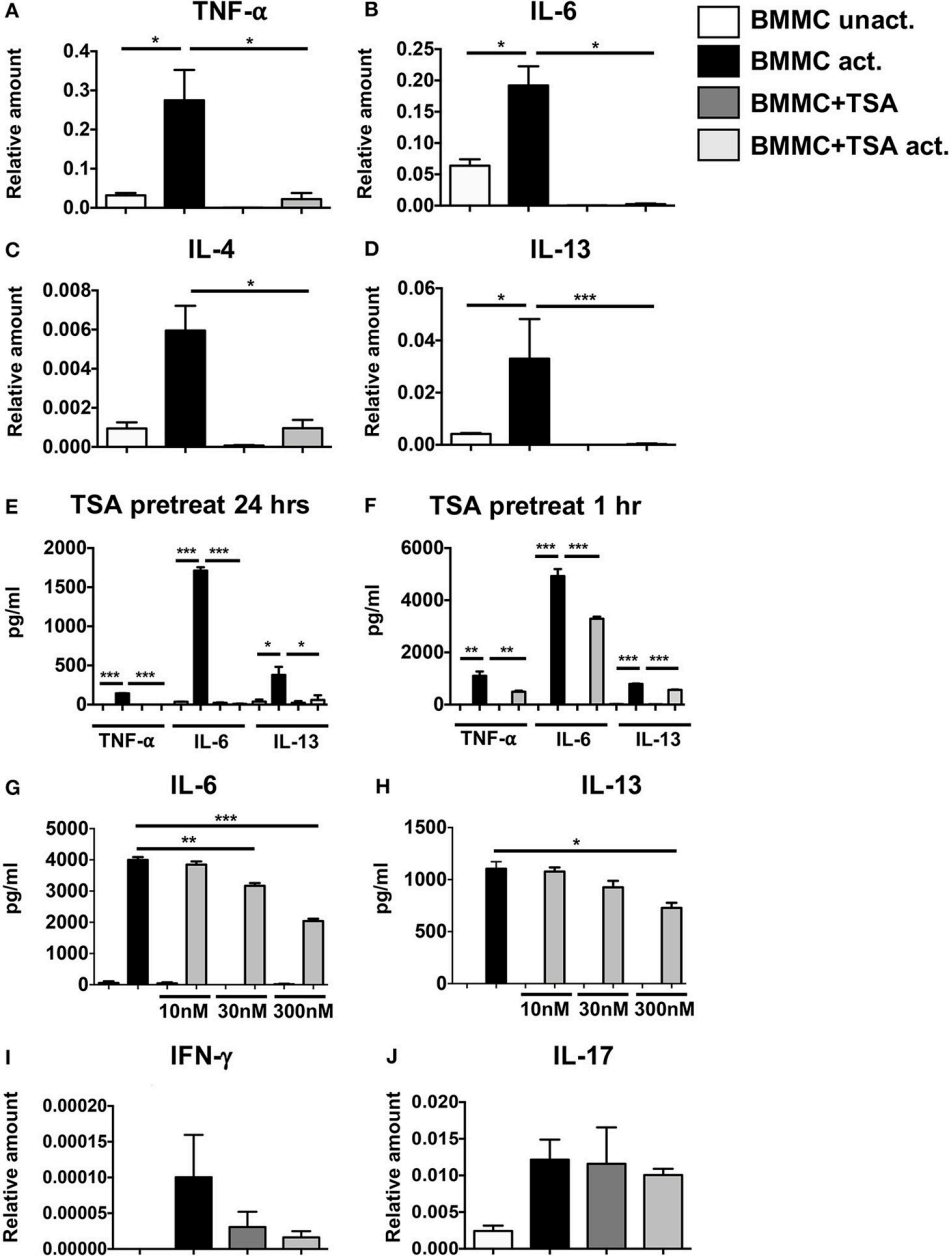
TSA suppresses cytokine gene expression and secretion in antigen-activated cells. BMMCs were pre-treated with 500 nM TSA or vehicle overnight. Some groups of cells were also simultaneously primed with 1μg/ml DNP-IgE. The next day, all cells were activated with 200 ng/ml DNP-BSA. **(A–D)** 1 h later, cells were collected for mRNA analysis. Transcripts relative to GAPDH are shown **(E)** Levels of cytokines secreted 12 h after activation are shown. **(F)** In other experiments, BMMCs were pre-treated with 500 nM TSA for 1 h prior to activation with antigen. Levels of cytokines secreted 12 h later are shown. **(G,H)** BMMCs were pre-treated overnight with varying doses of TSA prior to IgE and antigen activation. Cytokines secreted 12 h later are shown. **(I,J)** mRNA transcripts in BMMCs pre-treated with 500 nM TSA overnight and activated with IgE and antigen. Data are representative of 5–6 independent experiments. *p < 0.01; **p < 0.001; ***p < 0.0001 by Students *t*-test.

IgE-induced mast cell activation plays a critical role in the development of anaphylactic symptoms to allergenic stimuli. To further examine the effects of TSA treatment on IgE-mediated activation of mast cells, BMMCs were cultured with rIL-3 and rSCF and activated with DNP-specific IgE and DNP-BSA. Challenge with DNP-BSA in IgE-primed BMMCs resulted in a robust induction of the genes for TNF-α, IL-6, IL-4, and IL-13 compared to unactivated controls (Figures 2A–D). Similarly the secretion of TNF-α, IL-6, and IL-13 was also significantly enhanced in IgE-activated BMMCs (Figure 2E). In contrast, pre-treatment with TSA for 24 or 1 h resulted in a significant suppression of both the expression and secretion of cytokines (Figures 2A–F and data not shown). A similar pattern of cytokine inhibition was also observed in freshly isolated peritoneal mast cells that had been pre-treated with TSA and activated with IgE and antigen, suggesting that the effects of TSA are not limited to BMMCs alone, but can also extend to connective tissue mast cells *in vivo* (Supplementary Figures 1A,B). To further determine whether the effect of TSA on BMMCs was dose-dependent, BMMCs were treated with increasing concentrations of TSA overnight, and its effects on IgE-mediated activation were assessed. Treatment with increasing concentrations of TSA demonstrated a dose-dependent suppressive effect on the secretion of IL-6 and IL-13 (Figures 2G,H). To further assess the effects of TSA on BMMC cytokine production, we also examined the expression levels of IFN-γ and IL-17 in BMMCs. Interestingly, the expression of both IFN-γ and IL-17 was detected in BMMCs, suggesting that they can produce Th1 and Th17 cytokines (Figures 2I,J). Treatment with IgE and antigen resulted in enhanced expression of both IFN-γ and IL-17 in BMMCs compared to unactivated controls. However, in contrast to the expression of TNF-α, IL-6 and IL-13, no changes in IFN-γ and IL-17 expression were observed in TSA-treated and IgE-activated BMMCs (Figures 2I,J). These data therefore suggest that TSA treatment can differentially modulate the production of cytokines by mast cells stimulated with IgE and antigen.

### TSA treatment attenuates mast cell degranulation and FcεRI expression

**Figure 3:**
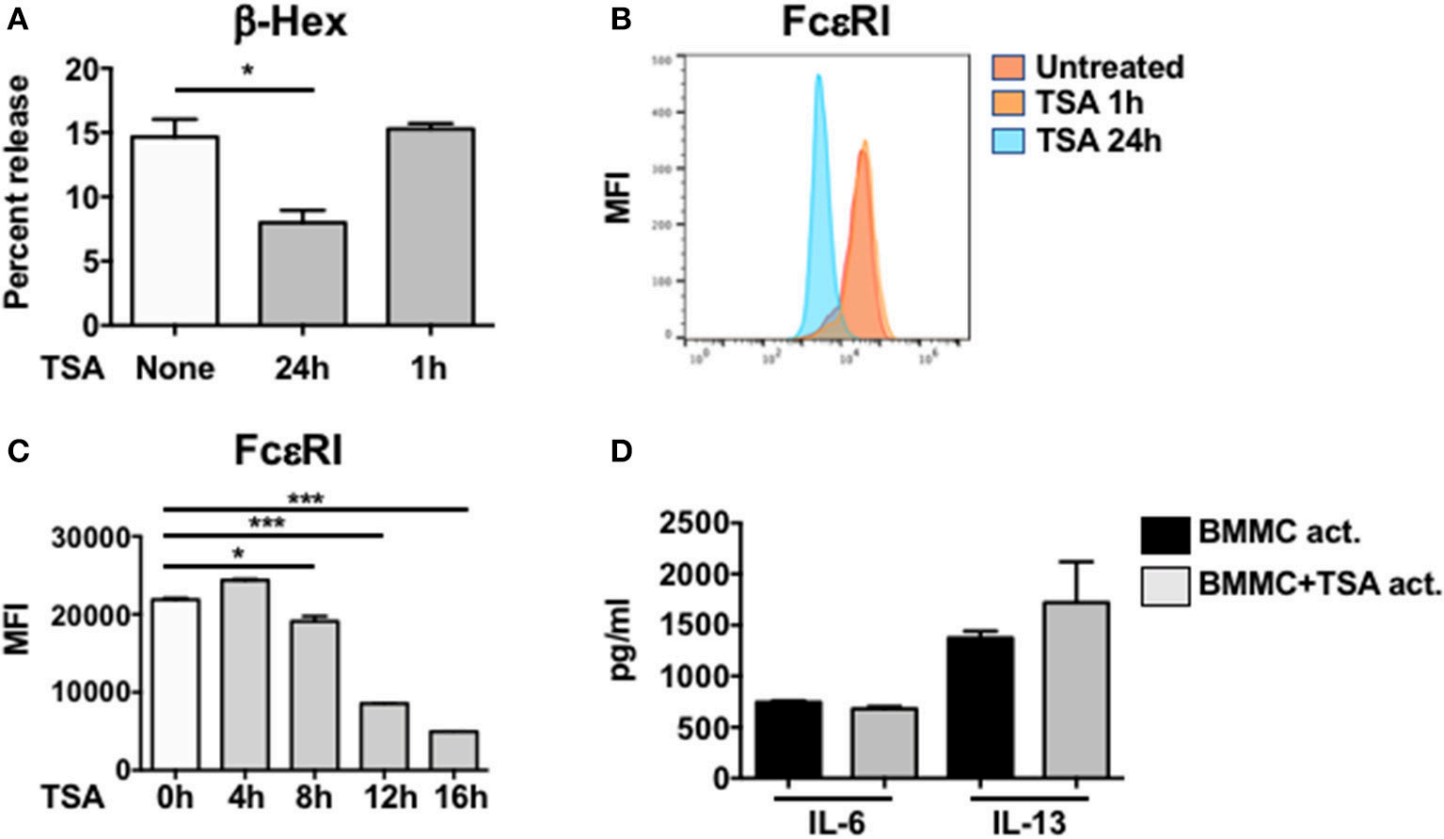
TSA attenuates mast cell degranulation and FcεRI expression. **(A)** BMMCs were treated with vehicle or 500 nM TSA for either 24 or 1 h prior to IgE-induced activation with DNP-BSA. β-hex levels were enumerated in cell supernatants and lysates. Percent release of β-hex is shown **(B)** BMMCs were treated with vehicle or 500 nM TSA for either 24 or 1 h. The expression of FcεRI was evaluated by flow cytometry. Mean fluorescence intensity (MFI) is shown. **(C)** Kinetics of FcεRI expression after TSA treatment is shown. **(D)** BMMCs were activated with DNP-IgE and antigen. One minute after activation, cells were treated with either vehicle or 500 nM TSA. Cytokine levels in supernatants were enumerated 12 h later. Data are representative of 2 independent experiments. *p < 0.01; ***p < 0.0001 by Students *t*-test.

Since pretreatment with TSA inhibited the production of proinflammatory cytokines from IgE-activated mast cells, suggesting that TSA can modulate the *de novo* synthesis of mast cell cytokines, we wondered whether it could also similarly regulate mast cell degranulation. Mast cell degranulation was assessed by examining the release of β-hexosaminidase (β-hex) in cell culture supernatants. As we had anticipated, while IgE and antigen-activated BMMCs exhibited increased β-hex release, 24 h pre-treatment with TSA significantly decreased the percent of β-hex release in activated mast cells, suggesting that TSA treatment can inhibit IgE-mediated degranulation of BMMCs (Figure 3A). Similar results were also obtained with freshly isolated peritoneal mast cells that had been treated with IgE and antigen (Supplementary Figure 1C). Surprisingly, however, 1 h pre-treatment with TSA did not result in any attenuation of BMMC degranulation (Figure 3A).

Since the IgE-mediated activation of mast cells is dependent on the expression of the high affinity IgE receptor, FcεRI on mast cells, we examined whether TSA treatment modulates the expression of this receptor in resting mast cells. BMMCs were cultured with rIL-3 and rSCF for up to 24 h in the presence or absence of TSA and the expression of FcεRI was assessed by flow cytometry at several time points. One and 4 h after treatment with TSA, no changes in mean fluorescence expression of FcεRI was observed in TSA-treated cells as compared with untreated controls (Figures 3B,C). However, a gradual decrease in FcεRI expression was observed starting at 8 h after treatment with significant suppression observed at 24 h, from an MFI of approximately 31,896–3,829 (Figures 3B,C). This suggests that one potential explanation for the decreased IgE-mediated degranulation of TSA-treated mast cells may be related to decreased IgE binding. However, it does not account for inhibition of cytokine production in IgE-activated mast cells, since cytokine suppression was observed in mast cells pretreated with TSA for both 1 and 24 h, suggesting that decreased IgE binding may only partly be responsible for the observed effects. To therefore assess whether TSA treatment prevented the *de novo* synthesis of mast cell cytokines after IgE-mediated activation had already occurred, we treated BMMCs with TSA immediately post-activation with IgE and antigen. To our surprise, TSA treatment after antigen-mediated activation had occurred had no effect on the release of cytokines, suggesting that the effects of TSA pretreatment are mediated by preventing the transcriptional activation of antigen-induced genes (Figure 3D).

### TSA pretreatment inhibits IL-33-mediated cytokine production in mast cells

**Figure 4:**
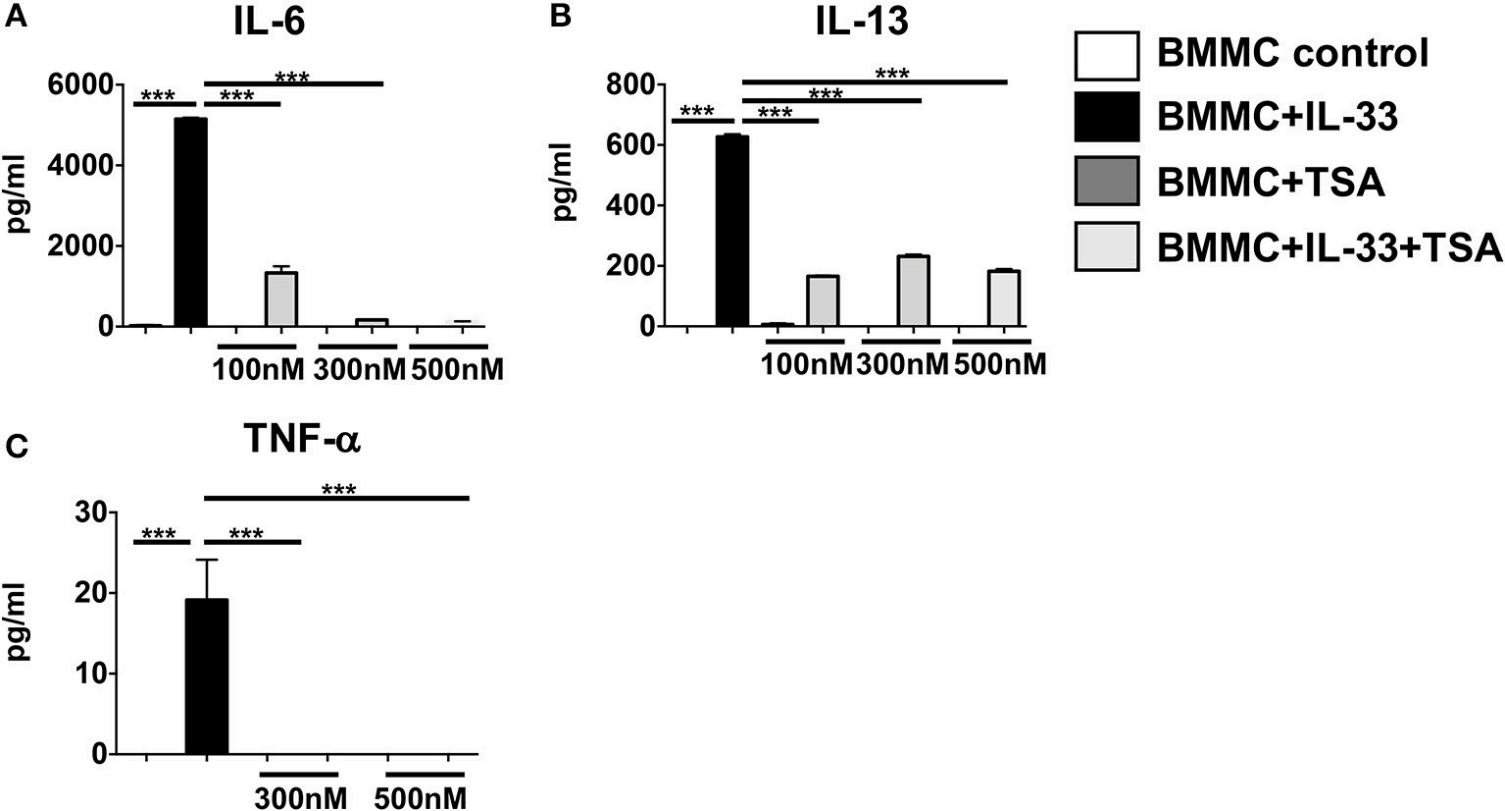
TSA suppresses IL-33-induced mast cell cytokines in a dose-dependent manner. **(A–C)** BMMCs were cultured with rIL-33 and varying doses of TSA or vehicle for 24 h. Levels of cytokines in supernatants were assessed by ELISA. Data are representative of 2 independent experiments. ***p < 0.0001 by Students *t*-test.

Our data suggest that TSA treatment modulates cytokine production in IgE-activated mast cells by regulating FcεRI expression. We were curious if TSA could also regulate cytokine production in mast cells stimulated independently of IgE. We therefore examined the effects of TSA on IL-33-stimulated mast cells, which is a potent inducer of mast cell cytokines. Treatment of mast cells with IL-33 resulted in the production of elevated levels of the cytokines IL-6, IL-13, and TNF-α (Figures 4A–C). In contrast, pre-treatment with TSA prior to IL-33 stimulation significantly decreased the levels of mast cell cytokines in a dose-dependent manner (Figures 4A–C). This suggests that TSA can also inhibit the production of mast cell cytokines stimulated with innate cytokines such as IL-33.

### Long-term TSA exposure reduces survival of BMMCs and induces apoptosis

**Figure 5:**
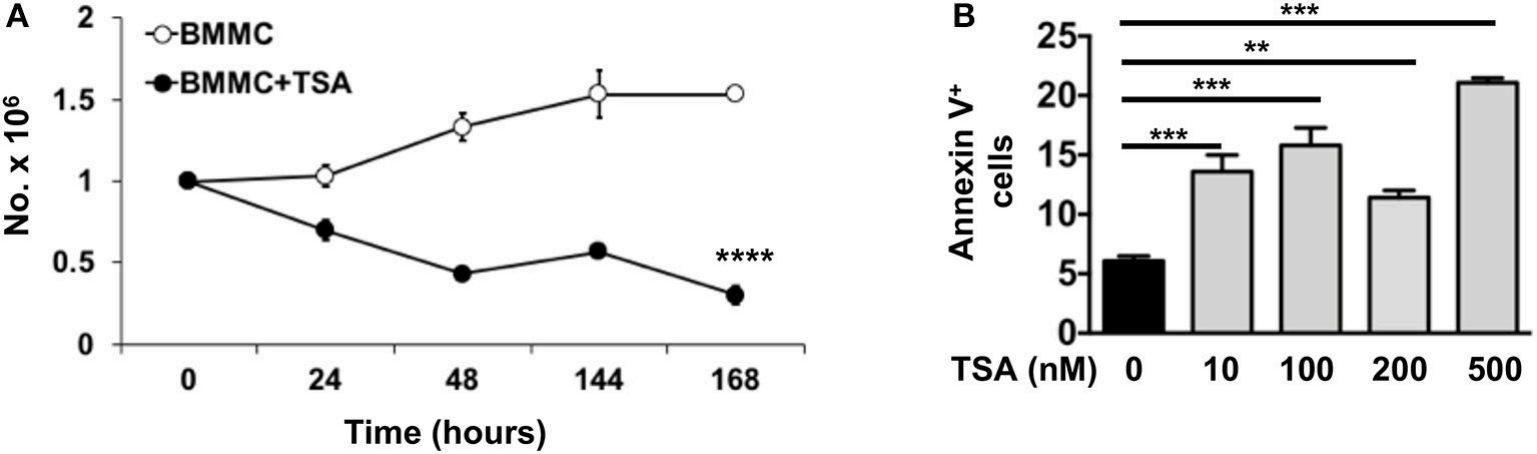
TSA induces apoptosis and inhibits mast cell proliferation. **(A)** BMMCs were cultured with rIL-3 and rSCF for 6 days in the presence or absence of 500 nM TSA. Cells were counted daily and live and dead cells were enumerated. **(B)** BMMCs were cultured with varying doses of TSA for 24 h and the expression of Annexin V was assessed by flow cytometry. Data are representative of 2 independent experiments. **p < 0.001; ***p < 0.0001 by Students *t*-test. ****p < 0.0001, by ANOVA.

We have previously shown that one of the mechanisms by which curcumin modulates mast cell function is to inhibit mast cell survival via the induction of apoptosis (28). To determine whether a similar mechanism may be involved in TSA-induced regulation of BMMCs, we examined the effects of TSA treatment on BMMC proliferation and survival. Continuous exposure to TSA over a period of 6 days resulted in a progressive decline in mast cell numbers, while BMMCs cultured with rIL-3 and rSCF proliferated normally over the same timeframe (Figure 5A). This suggested that TSA may induce apoptosis in BMMCs as has been described in other cell types similarly exposed to TSA (42, 60, 61). Further examination of BMMCs treated with TSA for 24 h demonstrated a dose-dependent increase in Annexin V signal via flow cytometry, suggesting that TSA is able to induce apoptosis in BMMCs after treatment for 24 h (Figure 5B).

### TSA treatment results in decreased NF-κB activation in IgE-activated BMMCs

**Figure 6:**
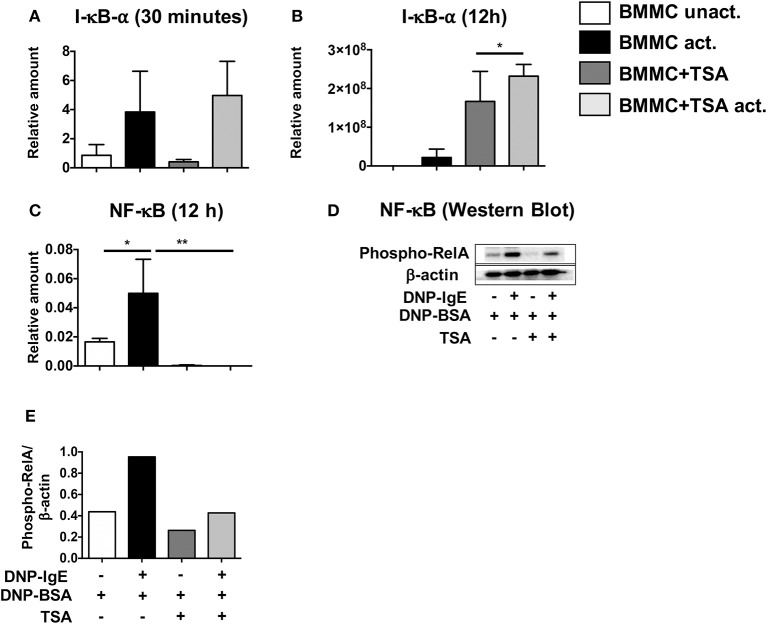
TSA induces I-κBα expression and inhibits NF-κB activation. **(A–C)** BMMCs were pre-treated with vehicle or 500 nM TSA overnight prior to activation with DNP-IgE and DNP-BSA. The expression of I-κBα and NF-κB was assessed by RT-PCR. **(D,E)** Western blot was performed on whole cell lysate protein extracts from BMMCs pre-treated with vehicle or TSA and activated with DNP-IgE and DNP-BSA. The amounts of phospho-relA and β-actin were assessed. Data are representative of 2 independent experiments. *p < 0.01; **p < 0.001 by Students *t*-test.

To further determine the cause of decreased mast cell cytokine production in activated mast cells, we next examined the NF-κB signaling pathway, a key regulatory pathway that is essential for the transcriptional activation of various cytokines. Since I-κBα is the negative regulator of NF-κB, we assessed whether TSA treatment would lead to transcriptional upregulation of I-κBα upon activation in IgE-activated BMMCs. Thirty minutes post activation with IgE and antigen, no changes in I-κBα expression were observed between TSA-treated and control BMMCs activated via IgE and antigen (Figure 6A). However, 12 h later, increased I-κBα expression was observed in TSA-treated and IgE-activated BMMCs compared to similarly treated controls (Figure 6B). Since I-κBα expression has been shown to fluctuate over time after cellular activation (62) and NF-κB acts as the transcription factor that directly regulates cytokine expression, we examined BMMCs for NF-κB expression after activation with DNP-IgE and antigen. At this time point, NF-κB expression was completely suppressed in TSA-treated BMMCs (Figure 6C). To further confirm the effects of TSA on NF-κB induction, we examined the levels of phospho-relA to determine the extent of NF-κB activation. The p65 (RelA) sub-unit of NF-κB plays a crucial role in the activation of NF-κB and its phosphorylation at Ser^276^ (phospho-relA staining) can be assessed by Western blot as we have previously described (28). Western blot analysis demonstrated increased levels of phospho-rel A in IgE-activated BMMCs compared to controls. In contrast, the level of phospho-relA was attenuated in similarly activated TSA-treated BMMCs (Figures 6D,E). These data therefore suggest that modulation of BMMC function by TSA may be mediated through altered activation of NF-κB.

### TSA treatment during oral allergen challenge inhibits diarrhea and mast cell activation during food allergy induction

**Figure 7:**
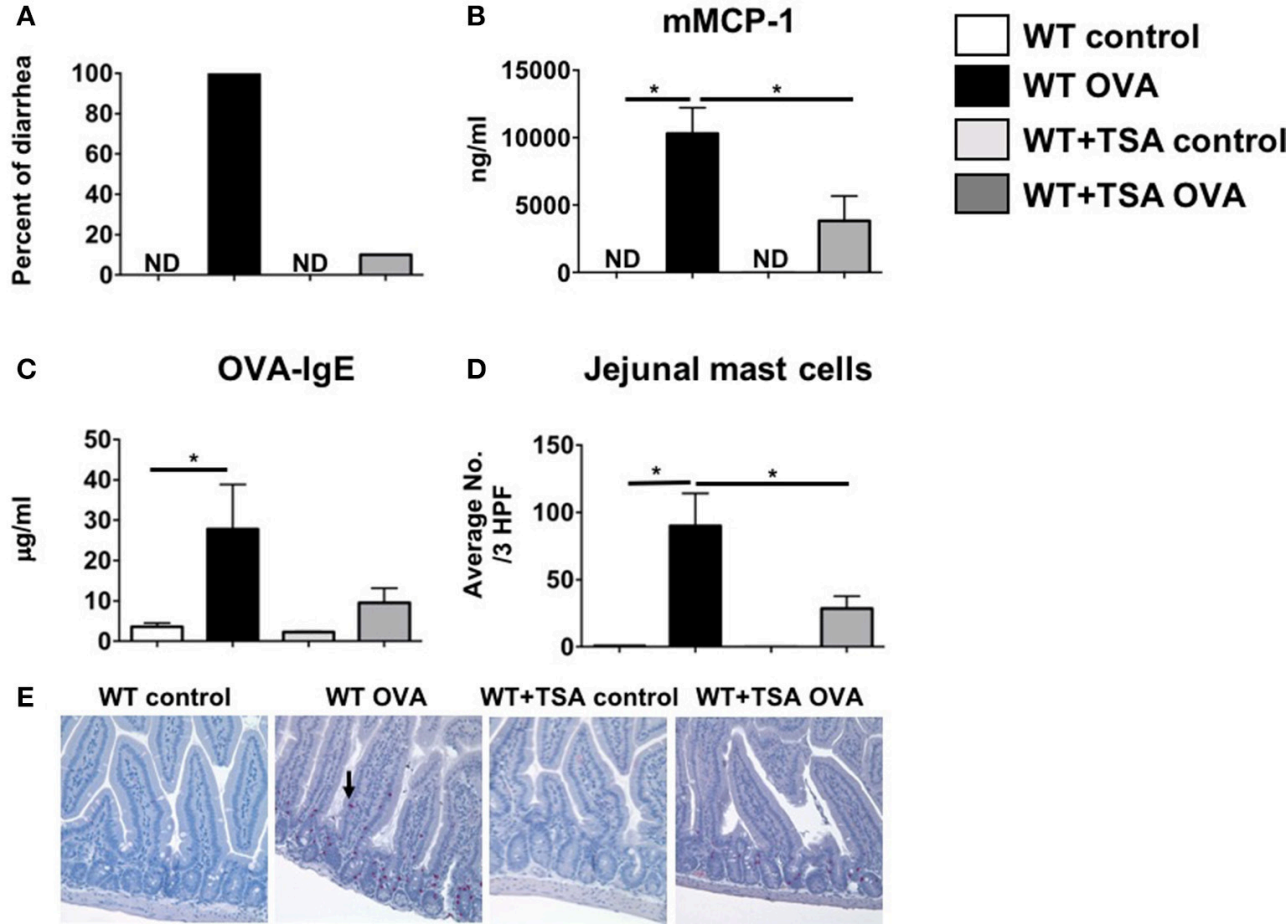
Treatment with TSA inhibits the development of allergic diarrhea and mast cell activation in OVA-sensitized and challenged mice. Mice were sensitized and challenged with OVA as described in Materials and Methods. Beginning 1 day prior to OVA challenges, mice were treated with vehicle or 75 μg TSA *i.p*. daily until sacrifice. **(A)** Percent of mice exhibiting diarrhea **(B)** serum mMCP-1 levels **(C)** serum OVA-IgE levels and **(D)** average numbers of jejunal chloroacetate esterase-positive mast cells/ 3 high powered fields (HPF) are shown. **(E)** Representative histological sections from individual mice are shown. Mast cells are indicated by an arrow. Data are representative of 3 independent experiments. *n* = 6 mice/group. *p < 0.01 by Students *t*-test. ND, not detected.

Since TSA treatment had a profound suppressive effect on mast cells in cell culture, we sought to determine whether it could similarly modulate mast cell function *in vivo* during the development of food allergy. Balb/c mice were sensitized and orally challenged with OVA as described in Materials and Methods and the development of food allergy was assessed. One hour after the final oral challenge with OVA, allergen-sensitized and challenged mice exhibited the development of profuse diarrhea as previously demonstrated (59). In contrast, similarly challenged TSA-treated animals exhibited a lower incidence of diarrhea overall in comparison with untreated animals (Figure 7A). Examination of sera for mast cell markers revealed elevated levels of murine mast cell protease-1 (mMCP-1) in OVA-challenged animals, suggesting increased mast cell activation (Figure 7B). Similarly, elevated levels of OVA-specific IgE were also present in the sera of allergic animals (Figure 7C). In contrast, the levels of both mMCP-1 and OVA-IgE were decreased in TSA-treated allergic animals (Figures 7B,C). Furthermore, the numbers of intestinal mast cells in TSA-treated mice were also decreased compared to untreated mice (Figures 7D,E).

**Figure 8:**
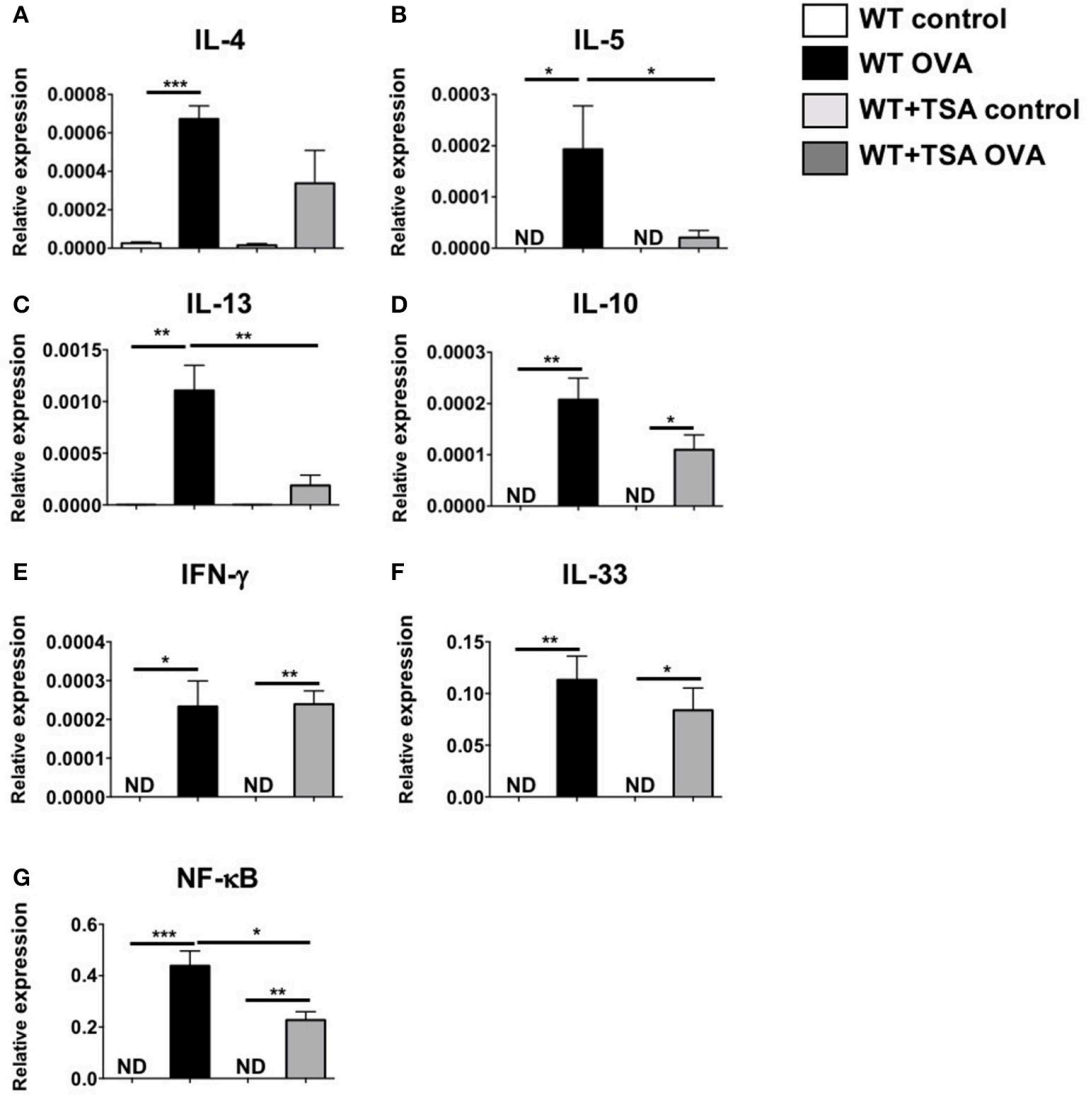
TSA modulates intestinal cytokine and NF-κB gene expression in allergic mice. **(A–G)** OVA-sensitized and challenged mice were sacrificed and RNA and cDNA were prepared from jejunal extracts. The expression of jejunal cytokines was assessed relative to GAPDH using Taqman probes. *n* = 6 mice/group. Data are representative of 2 independent experiments. *p < 0.01; **p < 0.001; ***p < 0.0001 by Students *t*-test. ND, not detected.

To further assess the effects of TSA treatment on modulation of cytokine production during food allergy, we examined the local and systemic production of Th2 type cytokines in allergic mice. As anticipated, examination of jejunal tissue for mRNA transcripts revealed the induction of a number of Th2-type cytokine genes in OVA-sensitized and challenged animals compared to controls (Figures 8A–F). In contrast, except for IFN-γ, decreased expression of these cytokines was observed in the intestines of most of the TSA-treated mice, suggesting that TSA is able to modulate cytokine production during food allergy development (Figures 8A–F). To further investigate the effects of TSA on NF-κB expression *in vivo*, we also examined jejunal tissue for NF-κB transcripts as a measure of transcriptional regulation during food allergy development. While OVA-sensitized and challenged mice exhibited increased levels of total NF-κB mRNA in jejunal tissue compared to control mice, the expression of NF-κB was significantly reduced in the intestines of similarly challenged and TSA-treated animals, confirming the results we had observed in cell culture with BMMCs (Figure 8G).

**Figure 9:**
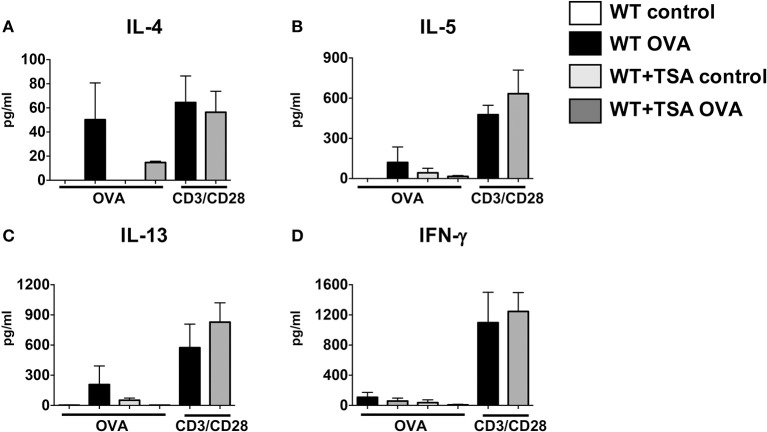
TSA modulates cytokine secretion in OVA-stimulated mesenteric lymph node (MLN) cells. **(A–D)** MLN cells were collected from experimental animals sensitized and challenged with OVA and stimulated for 96 h with either OVA or anti-CD3 and anti-CD28 as described in section Materials and Methods. Culture supernatants were evaluated for secretion of cytokines. *n* = 6 mice/group. Data are representative of 2 independent experiments.

To further confirm the effects of TSA on modulation of cytokine production by T cells, we examined the production of cytokines in mesenteric lymph node (MLN) cells from experimental animals in response to stimulation with OVA or T cell agonists. In comparison to controls, exposure to OVA induced the production of Th2 cytokines in MLN cells from WT OVA mice that had been sensitized and challenged with OVA (Figures 9A–D). In contrast, MLN cells from several OVA-sensitized and challenged TSA-treated animals exhibited a tendency toward lower levels of cytokine production overall compared with untreated animals (Figures 9A–D). Interestingly, however, TSA treatment had no effect on modulating the potential for cytokine production, since polyclonal activation of T cells in both groups resulted in equivalent levels of cytokine production (Figures 9A–D). These data therefore suggest that TSA can regulate the expression of cytokines in intestinal tissue and differentially modulate the production of cytokines by mast cells and T cells during food allergy development.

## Discussion

Epigenetic regulation of immune cell behavior is becoming increasingly accepted as a likely mechanism by which immune cell subsets mediate responses to widely differing stimuli. Mast cells, like many other immune cell types are extremely versatile and perform numerous functions contributing to the development of both innate and adaptive immune responses (63–66). As cells that can be rapidly activated during immune responses, they respond to diverse stimuli including alarmins such as IL-33, pathogen components such as TLR ligands, and antigen engagement *via* antibodies such as IgE and IgG. It is therefore extremely likely that the activation and function of mast cells during immune responses is regulated *via* epigenetic modifications induced by environmental exposure such as dietary antigens (67, 68).

In this report, we show for the first time that the inhibition of HDAC enzymes has a significant effect on mast cell activation and function during food allergy. Furthermore, we demonstrate that HDAC inhibition profoundly inhibits mast cell activation, degranulation and cytokine production *in vitro* in response to both IgE-dependent and independent stimuli, suggesting that modification of histone acetylation may be a putative mechanism involved in modulating mast cell function.

TSA is a broad spectrum HDACi that has several pleiotropic effects on cellular gene expression, including both the upregulation and downregulation of genes involved in cellular functions. Examination of TSA in a number of disease models suggests that in general it has beneficial effects on cellular function, promoting the downregulation of chronic inflammation and the induction of apoptosis and anti-oxidative pathways (49, 52, 54, 57, 69–73). This has resulted in the FDA approval of several HDACi (not including TSA) as therapeutic agents, as well as further pursuit of the mechanisms by which HDACi promote or inhibit cellular function (33, 42, 44).

We have previously shown that frequent ingestion of curcumin, the active ingredient of the curry spice turmeric, results in the suppression of mast cell responses and the attenuation of mast cell-mediated experimental food allergy (28). Several studies have suggested that the effects of curcumin *in vivo* are mediated via epigenetic modulation of HDACs as well as HATs, prompting us to investigate whether the observed effects of curcumin on mast cells in our previous study were similarly induced *via* modification of histone acetylation (31). Emerging evidence from a number of studies suggests that both the expression of genes governing immune cell differentiation as well as the induction of allergic sensitization may be epigenetically modulated, further warranting examination of the effects of TSA treatment in our model of food allergy (25, 26, 74). Lastly, while some studies examining the effects of histone deacetylase inhibition on T cell and ILC2-mediated allergic inflammation have been published, its effects on mast cell-mediated allergic responses have not yet been examined (51, 52, 54, 55).

Examination of the effects of TSA in animal models of allergic airway disease (AAD) have yielded mixed results. TSA treatment in models of both acute and chronic AAD resulted in inhibition of airway inflammation, cytokine production, collagen deposition, mucus production and airway hyperresponsiveness (AHR) (52, 55). In contrast, another study reported that TSA treatment inhibits AHR, but not airway inflammation in a model of asthma (54). The effects of TSA in this model were induced by blocking calcium mobilization and inhibiting intracellular calcium release in airway smooth muscle cells. More recently, Toki et al. and Thio et al. demonstrated that TSA also inhibits ILC2-mediated and IL-33-dependent innate allergic inflammation (51, 58).

In this study, we sought to examine the effects of TSA treatment on mast cells in both cell culture as well as *in vivo* during the development of food allergy. Our data demonstrate that TSA can modulate the function of both resting mast cells, as well as mast cells activated *via* IgE and non-IgE pathways. A previous study suggested that TSA may have dichotomous effects on mast cells, inhibiting IL-6 production after IgE-mediated activation, but not when stimulated with LPS (75). In contrast, we demonstrate that the inhibitory effects of TSA are conferred irrespective of the type of stimulus and can be induced in both activated as well as resting mast cells. This is further corroborated by our observations demonstrating that while downregulation of FcεRI expression by TSA may partly account for the observed decreases in cytokine production, cytokine production is also inhibited in IL-33-stimulated mast cells, suggesting that the effects of TSA are mediated independently of the IgE pathway. Interestingly, however, TSA treatment immediately after IgE-mediated activation had occurred resulted in the reversal of cytokine inhibition, suggesting that the effects of TSA may be mediated by increasing the expression of an upstream negative regulator of transcription such as I-κBα, which is turned on soon after antigen-induced cross-linking has occurred.

Treatment with TSA also resulted in the apoptosis of mast cells over time and decreased their proliferation and survival, as has been observed with other cell types such as eosinophils and neutrophils (61). The anti-proliferative effects of TSA may partly contribute to the decreased cytokine production in activated mast cells, but it does not explain the complete suppression of cytokine production as observed in Figures 1–4, since there are still a significant number of nonapoptotic cells after 24 h of treatment with TSA and we observe significant downregulation of cytokines as early as 1 h after treatment.

Since we had previously shown that the effects of curcumin in our model of food allergy were mediated *via* inhibition of NF-κB activation, we examined the effects of TSA treatment on the induction of I-κBα and NF-κB, hypothesizing that TSA treatment may enhance the expression of I-κBα in activated mast cells. Examination of BMMCs 30 min after IgE-mediated activation demonstrated equivalent expression of I-κBα in both untreated and TSA-treated cells. However, this is not surprising since the expression of I-κBα is regulated through complex interactions with NF-κB in nuclear and cytoplasmic compartments involving an inducible autoregulatory pathway which results in increased I-κBα induction by activated NF-κB (46, 76, 77). As such, further examination several hours later revealed a significant increase in expression of I-κBα in TSA-treated cells compared with untreated controls. Taken together with the decreased NF-κB expression and phospho-relA levels in BMMCs, this suggests that TSA may modulate BMMC function by altering I-κBα transcription and NF-κB transcription and activation.

The suppressive effects of TSA on mast cells were also observed *in vivo* during the development of food allergy. TSA treatment during the challenge phase resulted in the inhibition of allergic diarrhea, the attenuation of mast cell activation and intestinal mast cell numbers, and the suppression of Th2 cytokine genes, suggesting that histone deacetylase inhibition can modulate mast cell function *in vivo* and ameliorate the mast cell-mediated effects of food allergy such as intestinal anaphylaxis. Furthermore, the protective effects of TSA were conferred during the challenge phase in already sensitized animals, suggesting that TSA can modulate the mast cell-dependent phase of the response and attenuate mast cell-mediated effects during acute episodes of allergic inflammation.

Further studies aimed at elucidating the mechanisms by which HDACi modulates mast cell function are warranted. In particular this includes examining the roles of upstream negative regulators of transcription such as I-κBα as well as genes involved in mitotic pathways such as the MAP kinase genes. The effects of TSA on mast cell responses during food allergy also need to be further examined. It will especially be important to assess the differential effects of TSA on mast cell homeostasis and function vis-à-vis its known epigenetic effects on histone acetylation as well as its effects on the recruitment and survival of mature mast cells during allergic responses. Furthermore, TSA is a pan-HDACi and potentially has a wide range of other effects. As such, examination of the effects of specific HDACs in modulating allergic inflammation will provide further insight into the epigenetic regulation of mast cell function. In this context, a recent study demonstrated therapeutic effects of both HDAC6 and HDAC8 inhibitors in a mouse model of asthma (78). Similarly, other studies have demonstrated that HDAC enzymes such as HDAC6 and HDAC8 have a number of effects on non-histone targets including α-tubulin, actin and HSP90 (79–82). Thus, inhibition of these proteins has the potential to modulate the cytoskeleton as well as cellular morphology, migration and cellular interactions, which may contribute to the observed effects.

In summary, our data demonstrate that HDAC inhibition by TSA has a profound inhibitory effect on the activation and function of mast cells both in cell culture and during the development of food allergy, suggesting that the activation of mast cells is epigenetically regulated and that exposure to epigenetic modulators, including dietary components can alter the outcome of allergic disease in sensitized patients.

## Ethics statement

This study was carried out in accordance with the recommendations of the Institutional Animal Use and Care Committee of Western New England University. The protocol was approved by the Institutional Animal Use and Care Committee.

## Author contributions

CM and SK conceived the study design and directed the project. DK, EK, JR, SP, CT, CD, JS-D, SS, SK and CM contributed to experiments and analyzed data. DK, SK and CM prepared figures and wrote the manuscript.

### Conflict of interest statement

The authors declare that the research was conducted in the absence of any commercial or financial relationships that could be construed as a potential conflict of interest.

## Supplementary material

Supplementary Figure 1 TSA suppresses cytokine production in peritoneal mast cells. Peritoneal lavage was isolated from naïve mice and pooled. Cells were incubated with DNP-IgE in the presence or absence of 500 nM TSA overnight. The next day, cells were activated with DNP-BSA and cultured with Brefeldin A for 6 h. **(A)** Intracellular cytokine staining was performed on mast cells and the percent of IL-13 and TNF-α producing cells were assessed. **(B)** The total numbers of cytokine producing cells relative to absolute numbers are shown. **(C)** β-hex activity in cell culture supernatants was assessed.
