# Supplementary figures and images for: Epigenetic Regulation via Altered Histone Acetylation Results in Suppression of Mast Cell Function and Mast Cell-Mediated Food Allergic Responses
Source: Front Immunol. 2018 Oct 23;9:2414. doi: 10.3389/fimmu.2018.02414 (PMC6206211; doi:10.3389/fimmu.2018.02414)

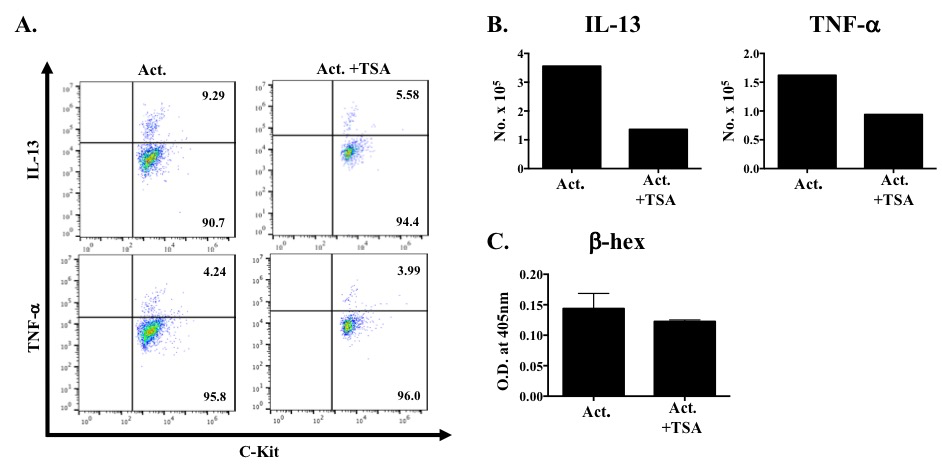

Supplement: Supplementary Figure 1 — TSA suppresses cytokine production in peritoneal mast cells. Peritoneal lavage was isolated from naïve mice and pooled. Cells were incubated with DNP-IgE in the presence or absence of 500 nM TSA overnight. The next day, cells were activated with DNP-BSA and cultured with Brefeldin A for 6 h. (A) Intracellular cytokine staining was performed on mast cells and the percent of IL-13 and TNF-α producing cells were assessed. (B) The total numbers of cytokine producing cells relative to absolute numbers are shown. (C) β-hex activity in cell culture supernatants was assessed. [file Image_1.TIFF]
